# Screen‐detected disordered eating and related traits in a large population sample of females in mainland China: China Health and Nutrition Survey

**DOI:** 10.1002/eat.23409

**Published:** 2020-11-15

**Authors:** Shuyang Yao, Ruyue Zhang, Laura M. Thornton, Christine M. Peat, Baiyu Qi, Shufa Du, Huijun Wang, Bing Zhang, Cynthia M. Bulik

**Affiliations:** ^1^ Department of Medical Epidemiology and Biostatistics Karolinska Institutet Stockholm Sweden; ^2^ Department of Psychiatry University of North Carolina at Chapel Hill Chapel Hill North Carolina USA; ^3^ Department of Nutrition University of North Carolina at Chapel Hill Chapel Hill North Carolina USA; ^4^ National Institute for Nutrition and Health, Chinese Center for Diseases Control and Prevention Beijing PR China

**Keywords:** body mass index, CHNS, eating disorders, epidemiology, women's health

## Abstract

**Objective:**

We describe the prevalence and sociodemographic factors associated with screen‐detected disordered eating and related traits in a population‐based sample of women in China. We also explored prevalence trends over time.

**Method:**

A total of 4,218 females aged 12–50 were sampled from 15 provinces as part of the China Health and Nutrition Survey (CHNS) in 2015. The SCOFF questionnaire screened for disordered eating and the selected questions from the Eating Disorders Examination‐Questionnaire measured dietary restraint, shape concerns, and weight concerns. Body mass index (BMI) was measured and sociodemographic factors captured urban/rural residence, age, ethnicity, income, education, marital status, and occupational status. We calculated the prevalence of screen‐detected disordered eating and related traits broadly and across several dimensions and compared prevalence estimates to 2009 and 2011 reports.

**Results:**

We detected 296 individuals who screened positive for disordered eating on the SCOFF (prevalence = 7.04%). Positive screens were associated with urban residence (*p* = .002) and higher education levels (*p* < .001). Scores on restraint, shape concerns, and weight concerns were all higher for individuals in urban versus village locations (all *p*'s < .001), and with higher BMI (*p* < .001) for shape and weight concerns. The prevalence of screen‐detected disordered eating increased numerically across 2009, 2011, and 2015.

**Discussion:**

The prevalence of screen‐detected disordered eating in mainland China was comparable to other populations worldwide obtained from a recent meta‐analysis. The distribution of disordered eating and related traits varied by several sociodemographic factors, which include age, BMI, urban/rural residence, education, and income, suggesting important directions for case detection and intervention in China.

## Graphical abstract

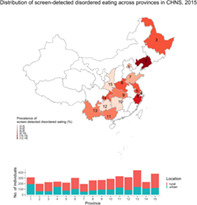

## 1 INTRODUCTION

Limited epidemiologic research on eating disorders (EDs) in Asia suggests comparable prevalence to other global regions (Pike & Dunne, 2015; Thomas, Lee, & Becker, 2016). China has a large and diverse population that has experienced rapid social and economic development over the last 40 years (Wen & Wolla, 2017). As the understanding of disease burden of EDs grows both globally and in Asia specifically (Hoek, 2016; Lee, 2000; Thomas et al., 2016), increased awareness and more comprehensive research are necessary to document ED‐related disease burden and to explore the association between socioeconomic factors and EDs in China (Sun, He, Fan, Chen, & Lu, 2020).

### 1.1 Epidemiology of eating disorders and related traits in China

The rise of EDs across Asia corresponded with broad and rapid economic and social transformation (Pike & Dunne, 2015; Wen & Wolla, 2017). Although China displayed somewhat lagged industrialization and modernization in Asia, it nonetheless reported an attendant rise in EDs (Pike & Dunne, 2015). Chen and Jackson (2008) sampled 1,320 females aged 12–22 years from academic settings in 10 Chinese cities and observed a prevalence of 2.3% for EDs using the Eating Disorder Diagnosis Scale (Stice, Telch, & Rizvi, 2000). Based on data collected in 2009 from The China Health and Nutrition Survey (CHNS), 6.3% adult females and 7.8% adolescent females had a screen‐detected ED (Watson et al., 2015). A later study (Tong et al., 2014) estimated the community prevalence in 8,521 female university students in Wuhan for clinical interview‐based diagnoses of anorexia nervosa (AN; 1.05%), bulimia nervosa (BN; 2.98%), and binge‐eating disorder (BED; 3.53%) to be comparable to that of their Western counterparts (e.g., 0.9%, 1.5%, and 3.5% among women in the US based on national survey data) (Hudson, Hiripi, Pope Jr., & Kessler, 2007). In terms of ED symptoms, Huon, Mingyi, Oliver, and Xiao (2002) sampled 1,246 schoolgirls across six Chinese cities and reported weight‐related concerns with 16% reporting being “very” or “extremely” concerned about their weight, despite >60% of individuals sampled being underweight.

### 1.2 Factors associated with eating disorders

Socioeconomic factors, such as urbanization (Gorrell, Trainor, & Le Grange, 2019; Hoek et al., 1995), employment status (Mulders‐Jones, Mitchison, Girosi, & Hay, 2017), education level (Striegel‐Moore & Bulik, 2007), income (Mitchison, Hay, Slewa‐Younan, & Mond, 2014), and civil status (Nevonen & Norring, 2004) have been associated with the risk of EDs in studies conducted primarily in Western populations, with mixed findings (Gard & Freeman, 1996; Schaumberg et al., 2017). A study from South Korea that evaluated associations among socioeconomic factors and EDs in an online survey of 6,943 adolescents indicated that disordered eating behaviors were associated with both high and low socioeconomic status (SES) in females (Lee et al., 2013). Chen and Jackson (2008), on the other hand, found that the prevalence of EDs was higher in adolescent and young adult females with higher household income (*p* < .001).

Urban/rural differences in ED symptoms have been observed in Chinese schoolgirls (Huon et al., 2002). Specifically, a lower proportion of girls from rural Hunan reported body dissatisfaction (desiring a lower BMI) and shape concerns, and a lower proportion of students had high scores on the Eating Attitudes Test (EAT‐26) (*n* = 266, 2.5% with high scores in EAT‐26) compared to two Southeast Chinese cities (Shenzhen: *n* = 286, 5.2% with high scores, and Hong Kong: *n* = 244, 9.74% with high scores). Feng and Abebe (2017) screened 466 middle school students in a rural area in the Northeast of China and found the prevalence of disordered eating behaviors to be 28.8%, which was higher than that reported in rural Hunan in the earlier study (Lee & Lee, 2000). However, in the absence of a comparison sample, it is not possible to determine the impact of urbanicity and geographic area on the presence of disordered eating behaviors. Further delineation of urban/rural differences is warranted.

EDs are present at all BMI levels, and on average, the BMI of individuals with AN is lower than that of individuals with BN, which is lower than that of BED (Schaumberg et al., 2017). Obesity could contribute to disordered eating behaviors, given that higher BMIs might induce body/shape dissatisfaction and precipitate unhealthy weight loss behaviors (da Luz et al., 2018). A survey of 2019 adolescent girls from seven cities in China illustrated a significant positive association between BMI and the Eating Disorder Inventory (EDI) scores of drive for thinness, body dissatisfaction, and bulimia (Fan et al., 2010). Survey data of adult females in the US also revealed increased risk for BN and BED among women who were overweight or obese compared with those with BMIs in the normal range (Duncan, Ziobrowski, & Nicol, 2017). However, neither the actual BMI nor the ideal BMI differed significantly between individuals with and without EDs that were detected by EDI screening and follow‐up interviews in female Chinese university students (Tong et al., 2014).

In the current study, we present prevalence estimates of screen‐detected disordered eating and related traits in the 2015 CHNS sample, and how the prevalence estimates were distributed across BMI levels and multiple sociodemographic domains (urbanicity, age group, ethnicity group, household income, education, marital status, and occupational status). We then compare prevalence estimates to the 2009 and 2011 waves of the CHNS to explore changes in the distributions over time. Although this work was primarily exploratory, we hypothesized that screen‐detected disordered eating and related traits would be more common in urban areas and that we could see increased prevalence across the three waves.

## 2 METHOD

CHNS, an ongoing longitudinal household‐based survey, was established in the 1980s to study the effects of the socioeconomic changes on nutrition and a variety of health outcomes, including eating behaviors and attitudes. The survey took place in 15 provinces and municipal cities, covering areas with different urbanization levels and varying substantially geographically (Du, Lu, Zhai, & Popkin, 2002; Zhang, Zhai, Du, & Popkin, 2014). CHNS has collected information about disordered eating in 2009, 2011, and 2015, affording the opportunity to explore sequential cross‐sectional overviews of the distributions of EDs in the population across a broad geographic distribution. CHNS was approved by both Institutional Review Boards at the University of North Carolina at Chapel Hill (UNC), Regional Ethical Review Board in Stockholm, and the Chinese Center for Disease Control and Prevention and the present study was approved by the Institutional Review Board at UNC.

### 2.1 Participants

The CHNS is an ongoing open cohort initiated in 1989 using a multi‐stage, random cluster sampling strategy to draw a sample with variation in geography, economic development, and public resources from China nationwide. The data were collected every 2–4 years from 1989 to 2015 in 10 waves. The most recent database released for research includes more than 30,000 individuals from over 7,200 households located in 216 communities.

From 2009 and onwards, ED questions were measured directly as part of the survey for females aged 11–41 years (2009 and 2011 waves) and 12–50 years (2015 wave). The provinces involved in wave 2009 are Guangxi, Guizhou, Heilongjiang, Henan, Hubei, Hunan, Jiangsu, Liaoning, and Shandong. Besides these provinces, wave 2011 was also conducted in Beijing, Chongqing, and Shanghai. Three more provinces including Shaanxi, Yunnan, and Zhejiang were added in the 2015 wave.

The present study uses data from all three waves with available eating disorder screening and examination items (2009, 2011, and 2015 waves), yielding a sample of *N* = 4,218 (2015 wave), *N* = 1,511 (2011 wave), and *N* = 1,133 (2009 wave) eligible participants.

### 2.2 Measures

#### 2.2.1 Demographics

Sociodemographic data including urban/rural residence (further categorized as urban/suburban/town/village), birth year (adolescent or adult), ethnicity (Han or other), education level (categorized as primary school degree, middle school degree, high school degree, college or above), marital status (ever married or not), and occupational status (presently working or not) were obtained through the individual survey from CHNS. Household income per capita (categorized as poorest, poorer, middle, richer, richest by quintiles) was obtained from the CHNS household survey (see Data Availability).

BMI (kg/m^2^) was computed from height and weight obtained from physical exams during the interviews. To make it more comparable with previous studies (Duncan et al., 2017), adults with valid BMI information were categorized into four groups: underweight (BMI < 18.5), normal weight (18.5 ≤ BMI < 24.0), overweight (24.0 ≤ BMI < 28.0), and obese (BMI ≥ 28.0) (Zhou, 2002). For adolescents, different BMI groups were defined according to the age‐ and sex‐specific BMI cut‐offs from the National Health Commission of China (http://wsbz.nhc.gov.cn/wsbzw/).

#### 2.2.2 Screen‐detected disordered eating

As with all large population studies that assess multiple domains, difficult choices must be made when balancing information accuracy and participant burden. The Chinese version of the SCOFF questionnaire (Leung et al., 2009; Morgan, Reid, & Lacey, 1999) was chosen because it is a widely used and very brief screen that addresses the core features of AN and BN. Additional questions from the Eating Disorder Examination‐Questionnaire (EDE‐Q v.6.0) (Fairburn & Beglin, 2008) (see below) were added to capture dimensions not covered by the SCOFF.

The procedures have been described in detail previously (Watson et al., 2015). The SCOFF has adequate psychometric properties (Kutz, Marsh, Gunderson, Maguen, & Masheb, 2019), although its sensitivity is somewhat lower in community samples, and has been found to have acceptable psychometric properties in a sample of Chinese secondary‐school students (Leung et al., 2009). Questions are included in Supplementary Material.

We defined and analyzed eating disorder patterns based on the answers to the SCOFF questionnaire (scored yes/no): screen‐detected disordered eating was defined as endorsing two or more of the five SCOFF items. Forty percent or more of valid responses were required for inclusion. We developed algorithms to identify patterns of SCOFF responses reflecting clinical presentations that were more typical of AN, BN, and BED. These algorithms are presented in Supplemental Material. Note, these algorithms do not provide a diagnosis and only index eating disorder‐like patterns.

#### 2.2.3 ED‐related traits

Seven questions from the Eating Disorder Examination‐Questionnaire (EDE‐Q v.6.0) (Fairburn & Beglin, 2008) assessed ED‐related traits in the individual survey in CHNS 2009, 2011, and 2015 waves. Administered items are listed in Table S1. Restraint scores were calculated by averaging ratings from the full EDE‐Q restraint subscale (Q6‐Q10). Shape concern was captured by Q11 and weight concern by Q12. The scores on this scale and these items range between 0 and 6. Higher scores indicate greater psychopathology. The EDE‐Q has been shown to have adequate psychometric properties (Fairburn & Beglin, 2008), and the individual items show a strong association to the underlying latent factor (Forsen Mantilla, Birgegard, & Clinton, 2017); however, the use of individual items to index subscales has not been tested and should be considered when interpreting results. The frequencies of each response category in three waves are provided in Table S2.

### 2.3 Statistical analysis

For categorical variables, cross‐tabulations and percentages for the prevalence of screen‐detected disordered eating with each sociodemographic variable (urban/rural residence, age [adult/adolescent], ethnicity, income, BMI, education, marital status, and occupational status) were computed for the 2015 wave. Adolescents (*n* = 370) were excluded from the analyses for education, marital status, and occupational status. Chi‐square tests were conducted to identify significant differences in screen‐detected disordered eating between groups for each sociodemographic variable. Cramer's Vs were reported as effect size indicators. Age was further categorized into 5‐year groups and the prevalence in each age group was explored in supplementary analysis.

Means and *SD* for the EDE‐Q measures (i.e., restraint, shape concern, and weight concern) were computed overall and separately for specific sociodemographic groups for the 2015 wave. *t* tests and ANOVA tests were used for comparing differences in the means of each score between sociodemographic groups. Eta‐squared were provided as effect size estimators.

To correct for multiple comparisons, we employed a modified Bonferroni approach, correcting for each of the eight sociodemographic domains tested (urban/rural, adult/adolescent, ethnicity, income, BMI, education, marital status, occupational status). *p* values <.00625 were considered to be significant. Further post‐hoc pairwise *t* test comparisons with Bonferroni adjustments were performed to test differences between levels for the significant omnibus Chi‐square tests or ANOVA.

To investigate the changes in the prevalence of any screen‐detected disordered eating over time, we performed a similar descriptive analysis to the 2009, 2011, and 2015 waves with age standardization using wave 2015 as reference. To enhance comparability, we further restricted the analysis to the nine provinces that were sampled in all three waves and estimated age‐and‐province‐standardized prevalence.

Data management was performed using SAS, version 9.4 (SAS Institute Inc, 2004) and analyses were performed using R, version 3.6.2.

## 3 RESULTS

### 3.1 Screen‐detected disordered eating in wave 2015

**FIGURE 1:**
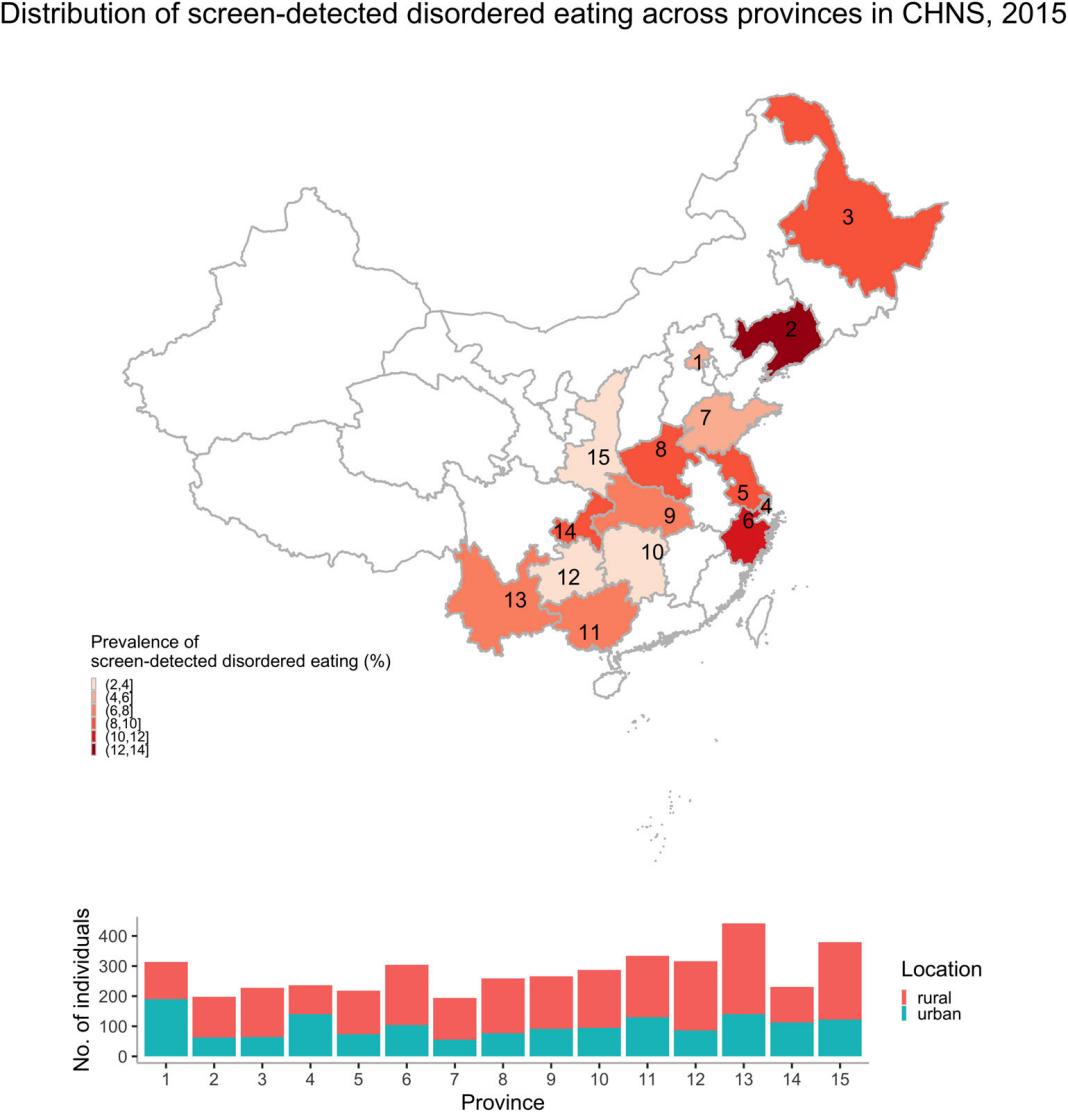
Distribution of screen‐detected disordered eating across provinces in CHNS, 2015. Province: 1=Beijing, 2=Liaoning, 3=Heilongjiang, 4=Shanghai, 5=Jiangsu, 6=Zhejiang, 7=Shandong, 8=Henan, 9=Hubei, 10=Hunan, 11=Guangxi, 12=Guizhou, 13=Yunnan, 14=Chongqing, 15=Shaanxi [Color figure can be viewed at wileyonlinelibrary.com]

The sample of 4,218 females with ED measures came from urban and rural areas in 15 Chinese provinces/municipalities. The average age (mean ± *SD*) of the study population was 35.64 ± 10.03 years with BMI (mean ± *SD*) of 23.35 ± 4.65 kg/m^2^. Using the SCOFF questionnaire, 296 individuals (prevalence = 7.04%) were determined to have screen‐detected disordered eating. The prevalence was highest in Liaoning, Zhejiang, and Chongqing (Figure 1). In terms of specific ED presentations, we detected 22 (0.52%) AN‐like, 28 (0.66%) BN‐like, and 84 (2.00%) BED‐like screen‐detected patterns. Prevalence did not significantly differ across ethnicity, income level, BMI groups, marital status, or occupational status (Table 1). In addition, prevalence did not differ between adolescents and adults. However, individuals aged 21–25 had the highest prevalence among all age groups, which was significantly higher than those below age 15 and those over age 36 (Table S3). The prevalence of any screen‐detected disordered eating was 11.24% in females aged 16–30. The prevalence of any screen‐detected disordered eating was higher in urban (10.16%) than in village (5.70%) communities (*p* < .001); urban prevalence was also numerically higher than that of suburban (7.51%, *p* = .27) and town (7.28%, *p* = .23) communities, although not statistically significant. The prevalence also differed significantly across education levels in adults (*p* < .001, Cramer's V = 0.100), with highest education level (college or above) associated with the highest prevalence (10.89%, *p* < .001 compared to other education levels). Notably, however, the number of missing values was high for BMI (*n* = 1,630), as well as for variables where we focused on adults, such as education level (*n* = 1,190) and occupational status (*n* = 1,014).

**TABLE 1:** Screen‐detected disordered eating based on SCOFF questions, CHNS wave 2015

	Total	Screen‐detected disordered eating (%)	Cramer's V	*p*‐value^a^
Overall	4,218 (12)^b^	296 (7.04)^c^		
Urban/rural residence			0.060	.002^*^
Urban	641 (1)	65 (10.16)		—
Suburban	908 (2)	68 (7.51)		.27
Town	715 (1)	52 (7.28)		.23
Village	1,954 (8)	111 (5.70)		<.001^§^
Adult/adolescent group			0.007	.68
Adolescent	370 (1)	24 (6.50)		
Adult	3,848 (11)	272 (7.09)		
Ethnicity group			0.014	.36
Han	3,742 (10)	267 (7.15)		
Other	453 (2)	27 (5.99)		
Missing	23	2 (8.70)		
Income level			0.041	.29
Poorest	603 (3)	35 (5.83)		
Poorer	604 (1)	39 (6.47)		
Middle	603 (4)	40 (6.68)		
Richer	604 (1)	50 (8.29)		
Richest	603 (2)	51 (8.49)		
Missing	1,200 (1)	81 (6.76)		
BMI group			0.036	.33
Underweight	187	10 (5.35)		
Normal	1,374 (3)	91 (6.64)		
Overweight	746 (5)	53 (7.15)		
Obesity	281 (2)	26 (9.32)		
Missing	1,630 (2)	116 (7.13)		
Education level			0.100	<.001^*^
Primary school	356 (3)	17 (4.82)		<.001^§^
Middle school	1,060 (3)	58 (5.49)		<.001^§^
High school	304 (2)	19 (6.29)		.056
College or above	938 (1)	102 (10.89)		—
Missing	1,190 (2)	76 (6.40)		—
Marital status			−0.036	.03
Ever married	2,587 (10)	166 (6.44)		
Not married or missing	1,261 (1)	106 (8.41)		
Occupational status			0.030	.11
Currently working	1,804 (4)	140 (7.78)		
Currently not working	1,030 (7)	63 (6.16)		
Missing	1,014	85 (6.52)		

*Note*: There were 12 individuals without a valid response. Adolescents were excluded in the analyses of education level, marital status, and occupational status.

Abbreviations: BMI, body mass index; CHNS, China Health and Nutrition Survey.

^§^ For variables with significant differences across levels (“Urban/rural residence” and “Education level”), we performed post‐hoc pairwise *t* tests with Bonferroni adjustments to test differences between levels. The sign § indicates significantly different from the reference level. The reference level for “Urban/rural residence” was “Urban,” and the reference level for “Education level” was “College or above,” the level of “Missing” was not compared.

^*^ Significant at the Bonferroni corrected level of *p* < .00625.

^a^ *p*‐Value from Chi‐square test.

^b^ The number in () is the number of individuals with missing values for screen‐detected disordered eating information; this is included in the total number.

^c^ The number in () is the row percentage of screen‐detected disordered eating.

### 3.2 ED‐related traits in wave 2015

#### 3.2.1 Restraint

Measured by the full restraint subscale of the EDEQ, restraint scores (overall mean = 0.10, *SD* = 0.43, range = 0–6) differed significantly between urban and rural residents (*p* < .001), with urban residents having the highest score (0.16), followed by suburban (0.11, *p* = .17 compared to urban), town (0.10, *p* = .07 compared to urban), and village locations (0.08, *p* < .001 compared to urban). No significant differences were observed for restraint on adolescent/adult groups, ethnicity, income level, BMI, education, marital status, or occupational status.

#### 3.2.2 Shape and weight concerns

Shape (overall mean = 0.44, *SD* = 1.40, range = 0–6) and weight concerns (overall mean = 0.30, *SD* = 1.19, range = 0–6) were each measured by one question from the EDE‐Q. Similar to screen‐detected disordered eating and restraint, mean shape and weight concerns were significantly higher in urban residents than in suburban and rural residents (Table 2).

**TABLE 2:** Mean (*SD*) of eating disorder‐related traits from the EDE‐Q subscales (restraint, shape concern, weight concern) from CHNS wave 2015

	Restraint			Shape concern			Weight concern		
Total number (number of missing)	4,218 (1)			4,218 (0)			4,218 (0)		
	**Mean (*SD*)**	**Eta‐squared**	***p*‐value**	**Mean (*SD*)**	**Eta‐squared**	***p*‐value**	**Mean (*SD*)**	**Eta‐squared**	***p*‐value** ^*^
Overall	0.10 (0.43)			0.44 (1.40)			0.30 (1.19)		
Urban/rural residence		0.0040	<.001^*^		0.0092	<.001^*^		0.0056	<.001^*^
Urban	0.16 (0.48)		—	0.74 (1.79)		—	0.50 (1.49)		—
Suburban	0.11 (0.48)		.17	0.46 (1.43)		<.001^§^	0.30 (1.19)		.009^§^
Town	0.10 (0.43)		.07	0.46 (1.42)		.001^§^	0.39 (1.36)		.55
Village	0.08 (0.39)		<.001^§^	0.32 (1.20)		<.001^§^	0.21 (1.00)		<.001^§^
Adult/adolescent group		0	.90		0	.86		0.0001	.46
Adolescent	0.10 (0.44)			0.45 (1.41)			0.35 (1.29)		
Adult	0.10 (0.43)			0.44 (1.40)			0.30 (1.18)		
Ethnicity group		0.0001	.49		0.0010	.05		0.0010	.04
Han	0.10 (0.44)			0.44 (1.40)			0.30 (1.19)		
Other	0.08 (0.37)			0.41 (1.38)			0.27 (1.13)		
Missing	0.09 (0.27)			1.17 (2.25)			0.96 (2.10)		
Income level		0.0006	.12		0.0013	.02		0.0019	.005^*^
Poorest	0.07 (0.35)			0.35 (1.27)			0.20 (0.94)		—
Poorer	0.08 (0.35)			0.30 (1.13)			0.20 (0.95)		1.00
Middle	0.10 (0.48)			0.44 (1.43)			0.31 (1.22)		.73
Richer	0.13 (0.49)			0.50 (1.50)			0.40 (1.37)		.02^§^
Richest	0.11 (0.38)			0.51 (1.48)			0.31 (1.14)		.95
Missing	0.11 (0.46)			0.48 (1.48)			0.36 (1.32)		
BMI group		0.0016	.01		0.0032	<.001^*^		0.0036	<.001^*^
Underweight	0.07 (0.27)			0.13 (0.77)		1.00	0.10 (0.64)		1.00
Normal	0.08 (0.39)			0.26 (1.07)		—	0.19 (0.92)		—
Overweight	0.10 (0.41)			0.54 (1.52)		<.001^§^	0.33 (1.22)		.04^§^
Obese	0.10 (0.36)			0.89 (1.92)		<.001^§^	0.54 (1.59)		<.001^§^
Missing	0.12 (0.49)			0.50 (1.51)			0.37 (1.33)		
Education level		0.0005	.18		0.0009	.06		0.0014	.02
Primary school	0.07 (0.39)			0.29 (1.16)			0.17 (0.84)		
Middle school	0.06 (0.27)			0.28 (1.12)			0.16 (0.87)		
High school	0.09 (0.46)			0.32 (1.18)			0.30 (1.20)		
College or above	0.17 (0.55)			0.72 (1.76)			0.49 (1.48)		
Missing	0.10 (0.43)			0.43 (1.40)			0.31 (1.23)		
Marital status		0.0010	.07		0.0024	.004^§^		0.0027	.003^§^
Ever married	0.09 (0.40)			0.39 (1.32)			0.26 (1.09)		
Not married or missing	0.12 (0.49)			0.54 (1.55)			0.39 (1.36)		
Occupational status		0	.73		0.0004	.22		0.0006	.12
Currently working	0.11 (0.44)			0.47 (1.44)			0.33 (1.22)		
Currently not working	0.07 (0.37)			0.32 (1.21)			0.20 (0.94)		
Missing	0.11 (0.46)			0.50 (1.50)			0.36 (1.33)		

*Note*: ANOVA were performed to test score differences across levels of each variable. For significant omnibus tests, pairwise *t* tests with Bonferroni adjustment were performed to test differences between the various levels and the referent group. There were 12 individuals without a valid response. Adolescents were excluded in the analyses of education level, marital status, and occupational status.

Abbreviations: BMI, body mass index; CHNS, China Health and Nutrition Survey; EDE‐Q, Eating Disorder Examination‐Questionnaire.

^*^ Significant at the Bonferroni corrected level of *p* < .00625.

^§^ For variables with significant differences across levels (“Urban/rural residence” and “BMI group”), we performed post‐hoc pairwise *t* tests with Bonferroni adjustments to test difference between levels. The sign § indicates significantly different from the reference level. The reference level for “Urban/rural residence” was “Urban,” and the reference level for “BMI group” was “Normal,” the level of “Missing” was not compared.

Scores for both shape and weight concerns differed across BMI levels (*p* < .001), with higher scores in overweight and obese groups compared to normal weight group (Table 2). Weight concern also differed across income groups, with the richer group being significantly higher than the poorest (*p* = .02), even though overweight and obesity were less prevalent in the richer (and richest) than in the poorer and poorest groups (Table 3).

**TABLE 3:** BMI distribution among different income levels, CHNS wave 2015

	Underweight (%)	Normal (%)	Overweight (%)	Obesity (%)	Total	*p*‐value^a^
Poorest	40 (7.80)^b^	250 (48.73)	164 (31.97)	59 (11.50)	513	
Poorer	47 (9.38)	255 (50.90)	131 (26.15)	68 (13.57)	501	
Middle	26 (5.26)	240 (48.58)	173 (35.02)	55 (11.13)	494	
Richer	34 (6.75)	286 (56.75)	128 (25.40)	56 (11.11)	504	
Richest	37 (7.25)	306 (60.00)	129 (25.29)	38 (7.45)	510	
Total	184	1,337	725	276	2,522	<.001*

*Note*: * indicates significant at the level of p < .05.

Abbreviation: CHNS, China Health and Nutrition Survey.

^a^ *p*‐value from Chi‐square test evaluating differences in BMI distribution across income levels.

^b^ The number in () is the row percentage.

Ever married adults scored lower than adults who had not been married or for whom marital information was not available (*p* = .004 for shape concern, *p* = .003 for weight concern). No significant differences emerged across adult/adolescent, ethnicity, education, or occupational status groups.

### 3.3 Prevalence trends

To evaluate whether the prevalence of screen‐detected disordered eating changed over time, we present the crude, age‐standardized, and age‐and‐province‐standardized prevalence of screen‐detected disordered eating in samples from the CHNS waves in 2009, 2011, and 2015 (Table 4). Without any restrictions on the samples, the crude prevalence of any screen‐detected disordered eating was 6.64%, 10.13%, and 7.04% in waves 2009, 2011, and 2015, respectively. However, the age‐standardized prevalence illustrated an increased trend across the three waves (5.80%, 6.12%, and 7.04% in waves 2009, 2011, and 2015, respectively), although this was not statistically significant. Since the three waves included different provinces, we further restricted the provinces in each wave to be the same (including Heilongjiang, Liaoning, Shandong, Henan, Hubei, Hunan, Jiangsu, Guangxi, and Guizhou). Age‐and‐province‐standardized prevalence in the province‐restricted samples was 5.88%, 5.99%, and 6.91% for the 2009, 2011, and 2015 waves, respectively, showing an increase in 2015 which was, however, not statistically significant (Table 4).

**TABLE 4:** Number, crude prevalence, and age‐ and province standardized prevalence of screen‐detected disordered eating in samples from the 2009, 2011, and 2015 CHNS waves, no restriction and restricted to the same province

		2009 wave	2011 wave	2015 wave
1. No restrictions	Total sample size (*N* of missing)	1,133 (19)	1,511 (1)	4,218 (12)
	*N* (crude prevalence^a^) screen‐detected disordered eating	74 (6.64%)	153 (10.13%)	296 (7.04%)
	Age‐standardized prevalence (95%CI) of screen‐detected disordered eating^b^	5.80% (3.60%, 8.02%)	6.12% (4.03%, 8.20%)	7.04% (6.24%, 7.84%)
	Difference (95%CI) in standardized prevalence compared to 2015 wave^d^	−1.23 (−3.59, 1.13)	−0.92 (−3.16, 1.32)	—
2. Restricted to the same provinces	Total sample size (*N* of missing)	1,133 (19)	993 (1)	2,312 (12)
	*N* (crude prevalence^a^) screen‐detected disordered eating	74 (6.64%)	80 (8.06%)	159 (6.91%)
	Age‐and‐province‐standardized prevalence (95%CI) of screen‐detected disordered eating^c^	5.88% (3.89%, 7.87%)	5.99% (4.55%, 7.44%)	6.91% (5.84%, 7.98%)
	Difference (95%CI) in standardized prevalence compared to 2015 wave^d^	−1.04 (−3.31, 1.23)	−0.92 (−2.73, 0.89)	—

*Note*: 1. When no restrictions were applied, the 2009 wave included females aged 11–39 years sampled from 9 provinces (detailed below); the 2011 wave included females aged 11–41 years sampled from the 9 provinces in the 2009 wave and three municipalities, including Beijing, Chongqing, and Shanghai; the 2015 wave included females aged 12–50 years from the 12 provinces/municipalities in the 2011 wave and three provinces, including Shaanxi, Yunnan, and Zhejiang. 2. To make the three waves more comparable, we limited the provinces to be the same across the three waves. The provinces included Heilongjiang, Liaoning, Shandong, Henan, Hubei, Hunan, Jiangsu, Guangxi, and Guizhou.

Abbreviations: CHNS, China Health and Nutrition Survey.

^a^ Crude prevalence was calculated as the number of screen‐detected disordered eating divided by the total number in the sample excluding missing data.

^b^ Age‐standardized prevalence in 1 was calculated using the 2015 wave (excluding missing) as reference, and age was grouped into 5‐year bins.

^c^ Age‐and‐province‐standardized prevalence in 2 was calculated using the 2015 wave with province restricted (excluding missing) as reference, and age was grouped into 5‐year bins.

^d^ Rate differences with 95% confidence interval were calculated to compare standardized prevalence between waves 2009, 2011, and 2015 with the 2015 wave as reference.

## 4 DISCUSSION

We described the prevalence of screen‐detected disordered eating and related traits and their distributions across a variety of sociodemographic domains in a large sample from mainland China. It is, to our knowledge, the largest study of its kind. In 4,218 females (aged 12–50 years) sampled across 15 Chinese provinces/municipalities in 2015, we observed a prevalence of 7.04% for any screen‐detected disordered eating using the recommended cut‐offs for the SCOFF questionnaire. The observed prevalence was even higher among females aged 16–30 with an estimate of 11.24%. Regarding specific ED presentations, the prevalence of screen‐detected AN‐, BN‐, and BED‐like patterns were 0.52%, 0.67%, and 2.00%, respectively.

Most available epidemiological studies on EDs in China have been based on student samples rather than the general population (Qian et al., 2013). The observed prevalence estimates in this general population survey were somewhat lower than the largest and most recent student survey (Tong et al., 2014). This is likely explained by our observation of significantly higher screen‐detected disordered eating in females with higher education levels, meaning we would expect a higher prevalence in purely university student samples. Our observed prevalence of any screen‐detected disordered eating in females aged 11–30 (Table S3) aligns with other Asian populations, such as South Korea [a prevalence of 14.8% for disordered eating behaviors was detected for school girls in an online survey in 2013, *n* = 3,467 (Lee et al., 2013)], Southeast Asia [11.5% university students were found at risk for EDs in a join sample from 5 Southeast Asian countries in 2018, *n* = 3,148, 63.3% female (Pengpid & Peltzer, 2018)], and Iran [a prevalence of 11.5% for any ED was reported in a community sample in 2012, *n* = 1,204, 54% female (Garrusi & Baneshi, 2012)].

The trend of increased prevalence of EDs has been observed globally—a recent meta‐analysis reviewing epidemiological studies on EDs published worldwide from 2000 to 2018 reported a lifetime prevalence of 8.4% (ranging between 3.3% and 18.6%) for any ED in females, with increasing trends in not only America, Europe, but also Asia (Galmiche, Déchelotte, Lambert, & Tavolacci, 2019). Specifically, a Japanese study illustrated increased prevalence for AN, BN, and EDNOS (i.e., eating disorder not otherwise specified) in a female student sample from 2002 to 2014 (Nakai, Nin, & Noma, 2014), and increased hospital visits due to EDs have been observed in Singapore from 2003 to 2010 (Lee et al., 2013). We also observed numerically increasing standardized prevalences of screen‐detected disordered eating across three waves of sampling in 2009, 2011, and 2015. However, the differences between the standardized prevalence estimates were not statistically significant, potentially due to smaller sample size in previous waves, especially after restricting the samples to the same provinces to enhance comparability.

Consistent with our hypothesis, of all variables we assessed, urban/rural residence was associated with one of the most significant differences in ED outcomes, although the effect size was small. Urban residents had higher risk of any screen‐detected disordered eating and higher scores on ED‐related traits than rural residents. Intriguingly, urban/rural residence could, in part, explain the observed province effect on the trend of disordered eating prevalence (Table 4). The crude prevalence in wave 2011 dropped from 10.13% in the unrestricted sample to 8.06% in the province‐restricted sample. However, the decrease in prevalence in wave 2015 was only from 7.04% in the unrestricted sample compared to 6.91% in the province‐restricted sample. The considerable drop of prevalence in wave 2011 after restricting the provinces could be partially explained by the fact that the restriction of provinces omitted three municipalities in 2011 (Beijing, Shanghai, and Chongqing, Figure S1), all with a relatively high urban/rural ratio. Accordingly, further excluding provinces with lower urban/rural ratio in 2015 (Zhejiang, Yunnan, and Shaanxi) could have neutralized the change in prevalence. This careful exploration of urban/rural differences reveals the complex association between the sociodemographic factors and the risk of EDs in China.

Our observations were in line with the hypothesis that urbanization contributes to the rise of EDs (Hoek et al., 1995; van Son, van Hoeken, Bartelds, van Furth, & Hoek, 2006). However, the mechanism behind this association is not clear or discernible from our data. Several potential risk avenues have been proposed including dietary practices (e.g., skipping meals and food preference) and internet and television access, which are possible correlates of urbanization (Becker, 2004; Peat et al., 2015; Watson et al., 2015). More detailed information about daily exposures in urban and rural situations are required in order to fully understand the phenomenon.

Open questions remain in the associations between income and disordered eating risk. Adding to the mixed literature (Koch et al., submitted; Schaumberg et al., 2017), our 2015 data only show a significant association between weight concern and income level, with no significant differences in screen‐detected disordered eating. Given that our weight concern measure was limited to one item, this result should be viewed with considerable caution and taken as an indication for further, more detailed investigation.

We also examined ED measures across BMI categories, divided according to norms for Chinese adolescents and adults. Higher BMI has been associated with increased risk of EDs and disordered eating behaviors in multiple populations and age ranges (Lee et al., 2013; McLean, Paxton, & Wertheim, 2010; Pengpid & Peltzer, 2018; Slevec & Tiggemann, 2011). In line with these studies, we also observed significantly higher scores for shape and weight concerns in individuals with higher BMI levels. In contrast, we did not observe significant differences on screen‐detected disordered eating (*p* = .33) or restraint (*p* = .01) across BMI categories, although the prevalence and means did increase numerically as BMI rose, suggesting that power may have limited our ability to detect significant differences.

Our results were split in terms of education levels with a clear increase in screen‐detected disordered eating as education level increased, but no significant differences in restraint or shape or weight concerns (although means increased numerically with increasing education levels). An association between eating disorders risk and higher education levels has been reported previously (Striegel‐Moore & Bulik, 2007). Recent research suggests that this association may not be due to environmental factors alone; genetic factors may also play a role as AN shows a strong significant genetic correlation with various measures of educational attainment (Watson et al., 2019).

The explanation for lower weight and shape concern scores in ever married versus never married women (and women with unknown civil status) is a matter of speculation. Previous studies have not shown differences on related measures such as body satisfaction by marital status (Friedman, Dixon, Brownell, Whisman, & Wilfley, 1999; Stevens & Tiggemann, 1998). One possibility is that marital status serves as a protective factor with partners serving a reassuring role (Birmingham, Cavallini, & Sgro, 2019); however, in the absence of more detailed information, we can only report this observation and recommend more detailed follow‐up in future studies.

Several limitations need to be acknowledged. First, our sample included only females. Surveys of males would be valuable to capture patterns of disordered eating in Chinese male population (potentially with gender‐relevant questions, such as drive for muscularity) (Lavender, Brown, & Murray, 2017). Second, the SCOFF does not specifically assess binge eating, so we may have underestimated the prevalence of this behavior. Third, our algorithms for AN‐, BN‐, and BED‐like patterns were not validated, so results should be viewed with caution. Fourth, the open cohort design means that the cohort is dynamic across time (i.e., the same individuals are not necessarily followed at each wave, and new provinces were added in later waves). Therefore, when evaluating trends, we restricted the analyses to provinces that participated in all three waves. The observed prevalence trend therefore only reflects the selected provinces. Fifth, we were unable to include the full weight and shape concern scales due to space limitations in the survey. However, the included individual items showed strong correlation with the underlying latent variable on each scale (0.80 and 0.84, respectively) in a population‐based sample of girls (Forsen Mantilla et al., 2017). The mean item scores in our sample (0.10[*SD* = 0.43] for EDE‐Q restraint, 0.44[1.40] for shape concern question, and 0.30[1.19] for weight concern question) were lower than scores in community/student samples from Western countries [e.g., Norway, *n* = 3,000, females aged 16–50 years (Rø, Reas, & Rosenvinge, 2012), Australia, *n* = 200, females aged 35–60 years (McLean et al., 2010)], other Asian populations [Japanese undergraduate females, *n* = 289, Restraint = 0.75[0.93], shape concern 2.67[1.50], weight concern 2.07 [1.41] (Nakai et al., 2014); and Iranian samples in Iran and in US (Abdollahi & Mann, 2001)], but also compared to scores in a smaller Chinese sample [aged 18–33, *n* = 195, 49.2% women, restraint: 4.58[6.38] (Tang, Forbush, & Lui, 2015)]. The discrepancy in scores might be due to translation or to the lack of consistency of EDE‐Q question inclusion across samples (Tang et al., 2015). Nevertheless, our scores had good internal consistency (standardized Cronbach's Alpha = 0.78 for restraint, 0.82 for all seven questions, Q6‐Q12, on ED‐related traits) and were consistent over time (Table S2). Although Chinese versions of EDE‐Q exist (He, Sun, & Fan, 2020; Lee et al., 2007; Tang et al., 2015), there are specific modifications and translational features and preference of the original EDE‐Q. A validated Chinese version of EDE‐Q is desirable for better comparability across studies. Sixth, due to the small size and range of our adolescent sample, we used BMI categories rather than BMI percentiles for this age group. Finally, our results are all based on self‐report. More nuanced interview‐based inquiries about eating disorders and related traits could yield a richer understanding of the evolution of eating disorders and related behaviors and attitudes in Chinese women.

In conclusion, in a large female sample in mainland China in 2015, we found comparable prevalence of screen‐detected disordered eating to more recent studies from other Asian populations and studies from Western countries. Our findings highlight the importance of screening for disordered eating and EDs in mainland China and increasing public recognition of these conditions. Both screen‐detected disordered eating and related traits varied across several sociodemographic factors, providing reference to identify risk groups for EDs. Among the explored factors, greater urbanicity showed the most consistent association, encouraging identification of specific risk factors associated with urbanization, such as stresses associated with urban living, living conditions, and eating habits. Other factors such as education level, income, BMI, and marital status suggest rich avenues of inquiry for more detailed explorations of risk factors for the development of eating disorders and related traits in China.

## CONFLICT OF INTEREST

C. M. B. reports: Shire (grant recipient, Scientific Advisory Board member); Idorsia (consultant); Pearson (author, royalty recipient). C. M. P. reports: Sunovion (Scientific Advisory Board member). All other authors report no conflicts of interest.

## DATA AVAILABILITY STATEMENT

Data from the China Health and Nutrition Survey (CHNS) are available at https://www.cpc.unc.edu/projects/china. The individual CHNS survey questionnaire is available at https://www.cpc.unc.edu/projects/china/data/questionnaires/C15Individual_Eng.pdf and the household survey questionnaire is available at https://www.cpc.unc.edu/projects/china/data/questionnaires/C15HH_Eng.pdf.

## Supporting information


**Appendix** **S1**: Supporting Information
