# Supplementary material for: Screen‐detected disordered eating and related traits in a large population sample of females in mainland China: China Health and Nutrition Survey
Source: Int J Eat Disord. 2020 Nov 15;54(1):24–35. doi: 10.1002/eat.23409 (PMC7855662; doi:10.1002/eat.23409)
Supplement: Supplementary file 1 — Appendix S1: Supporting Information [file EAT-54-24-s001.docx]

**Supplementary Material**

**Yao et al. Screen-detected disordered eating and related traits in a large population sample of females in mainland China: China Health and Nutrition Survey**

SCOFF questions 2015 (English):

Disordered eating (for females aged 12-49 years)

Q1. Do you make yourself Sick because you feel uncomfortably full?

Q2. Do you worry that you have lost Control over how much you eat?

Q3. Have you recently lost more than 6 kg (One stone) in a three-month period?

Q4. Do you believe yourself to be Fat when others say you are too thin?

Q5. Would you say that Food dominates your life?

Response options for each item were ‘yes’ and ‘no.’ (For Q3, if the loss of weight was due to disease, operation, or childbirth, please select ‘no’.)

SCOFF questions 2015 (Chinese):

三十四、进食失调 (12-49岁的女性)

1. 你有没有用催吐的方式来缓解因吃得过饱而引起不适？

0 没有

1 有

9 不清楚

2. 你有没有为自己进食失去控制而产生过担心？

0 没有

1 有

9 不清楚

3. 最近三个月内，你的体重减轻有没有超过6.35公斤(12.7 斤)？

*因疾病、手术、分娩等原因造成体重减轻者，应选“0”。

0 没有

1 有

9 不清楚

4. 如果别人说你太瘦，你是不是仍然觉得自己过胖？

0 没有

1 有

9 不清楚

5. 你是不是觉得食物可以控制你的行为和情绪？

0 没有

1 有

9 不清楚

Screen-detected eating disorder symptom patterns:

Screen-detected AN-like pattern: defined as ‘yes’ to both Q3 and Q4 (plus any other endorsed SCOFF items)

Screen-detected BN-like pattern: defined as ‘yes’ to both Q1 and Q2 (plus any other endorsed SCOFF items)

Screen-detected BED-like pattern: defined as ‘yes’ to both Q2 and Q5 and ‘no’ to Q1 (plus any other endorsed SCOFF items)

**Supplementary Tables**

Table S1. Eating Disorder Examination Questionnaire (EDE-Q v.6.0) items and the corresponding subscales administered in the China Health and Nutrition Survey, waves 2009, 2011, 2015.

| Subscale | Item |
| --- | --- |
| Restraint | Q6. Have you been deliberately trying to limit the amount of food you eat to influence your shape or weight? |
|  | Q7. Have you gone for long periods of time (8 hours or more) without eating anything in order to influence your shape or weight? |
|  | Q8. Have you tried to avoid eating any foods which you like in order to influence your shape or weight? |
|  | Q9. Have you tried to follow definite rules regarding your eating in order to influence your shape or weight; for example, a calorie limit, a set amount of food, or rules about what or when you should eat? |
|  | Q10. Have you had a definite desire to have an empty stomach with the aim of influencing your shape or weight? |
| Shape | Q11. Have you felt fat? |
| Weight | Q12. Have you had a strong desire to lose weight? |

Each item is prefaced with “On how many of the past 28 days…” Response options for each item were ‘no’, ‘1-5 days’, ‘6-12 days’, ’13-15 days’, ’16-22 days’, ’23-27 days’, and ‘daily’ and correspond to scores 0-6, respectively.

Table S2. Mean (SD) of eating disorder-related traits from the EDE-Q subscales restraint, shape concern, weight concern) from CHNS waves 2009, 2011, and 2015

|  | **Subscale** |  | **2009** | **2011** | **2015** |
| --- | --- | --- | --- | --- | --- |
| No limit | Restraint | N | 1110 | 1509 | 4217 |
|  |  | Mean (SD) | 0.098 (0.462) | 0.156 (0.553) | 0.101 (0.430) |
|  | Shape concern | N | 1111 | 1509 | 4218 |
|  |  | Mean (SD) | 0.435 (1.448) | 0.681 (1.767) | 0.439 (1.401) |
|  | Weight concern | N | 1111 | 1509 | 4218 |
|  |  | Mean (SD) | 0.324 (1.274) | 0.571 (1.677) | 0.304 (1.193) |
| Limit to same age range and provinces | Restraint | N | 1079 | 973 | 937 |
|  |  | Mean (SD) | 0.099 (0.467) | 0.122 (0.486) | 0.099 (0.407) |
|  | Shape concern | N | 1080 | 973 | 937 |
|  |  | Mean (SD) | 0.447 (1.467) | 0.532 (1.572) | 0.419 (1.370) |
|  | Weight concern | N | 1080 | 973 | 937 |
|  |  | Mean (SD) | 0.333 (1.291) | 0.429 (1.469) | 0.315 (1.198) |

We present frequency of response and scores (mean and standard deviation, SD) for both the full sample in the three waves (No limit) and limited to the same age range and provinces for better comparability.

Age range was limited to the intersection of the three waves: 12-35 years; provinces were limited to the intersection of the three waves, i.e., the provinces in the 2009 wave: Heilongjiang, Liaoning, Shandong, Henan, Hubei, Hunan, Jiangsu, Guangxi, and Guizhou.

Table S3. Age-specific prevalence of screen-detected disordered eating in the China Health and Nutrition Survey wave 2015.

| Age group | Number of individuals in the age group | Number of screen-detected disordered eating (prevalence %) | p-value |
| --- | --- | --- | --- |
| 11-15 | 255 | 11 (4.31) | 0.004* |
| 16-20 | 160 | 20 (12.50) | 1.00 |
| 21-25 | 284 | 36 (12.68) | - |
| 26-30 | 526 | 53 (10.08) | 1.00 |
| 31-35 | 598 | 45 (7.53) | 0.14 |
| 36-40 | 713 | 48 (6.73) | 0.02* |
| 41-45 | 928 | 48 (5.17) | <0.001* |
| 46-50 | 742 | 35 (4.72) | <0.001* |

We grouped age into 5-year bins. The overall p-value for testing prevalence differences across age groups is <0.001. p-values presented in the table are from pairwise t-test comparing each age group to the referent (age group 21-25) after Bonferroni adjustment for multiple comparisons.

* indicates significant difference.

Supplementary Figure

Supplementary Figure 1. Number/% of participants in urban and rural residences by provinces included in each wave

Legend:

Wave 2015: urban: 687/37.6%; rural: 1141/62.4%; after limiting provinces urban: 305/32.5%; rural: 632/67.5% (numbers exclude provinces added in 2015 compared to 2011: Shaanxi, Yunnan, Zhejiang).

Wave 2011: urban: 643/43.2%; rural: 846/56.8%; after limit provinces urban: 342/35.1%; rural: 633/64.9% (numbers exclude provinces added in 2011compared to 2009: Beijing, Chongqing, Shanghai)

Wave 2009: urban: 381/34.5%; rural: 723/65.5%
